# Sanguinarine inhibits epithelial–mesenchymal transition via targeting HIF-1α/TGF-β feed-forward loop in hepatocellular carcinoma

**DOI:** 10.1038/s41419-019-2173-1

**Published:** 2019-12-09

**Authors:** Qi Su, Mengying Fan, Jingjing Wang, Asmat Ullah, Mohsin Ahmad Ghauri, Bingling Dai, Yingzhuan Zhan, Dongdong Zhang, Yanmin Zhang

**Affiliations:** School of Pharmacy, Health Science Center, Xi’an Jiaotong University, Xi’an, 710061 P.R. China

**Keywords:** Pharmacology, Chemotherapy

## Abstract

Epithelial–mesenchymal transition (EMT) plays a crucial role in hepatocellular carcinoma (HCC) progression. Hypoxia and excessive transforming growth factor-β (TGF-β) have been identified as inducers and target for EMT in HCC. Here, we show hypoxia inducible factor-1α (HIF-1α) and TGF-β form a feed-forward loop to induce EMT in HCC cells. Further mechanistic study indicates under both hypoxia and TGF-β stimulation, Smad and PI3K-AKT pathways are activated. We show sanguinarine, a natural benzophenanthridine alkaloid, impairs the proliferation of nine kinds of HCC cell lines and the colony formation of HCC cells. In hypoxic and TGF-β cell models, sanguinarine inhibits HIF-1α signaling and the expression of EMT markers, translocation of Snail and activation of both Smad and PI3K-AKT pathways. Sanguinarine could also inhibit TGF-β-induced cell migration in HCC cells. In vivo studies reveal that the administration of sanguinarine inhibits tumor growth and HIF-1α signaling, inhibits the expression changes of EMT markers as well as Smad and PI3K-AKT pathway proteins. Our findings suggest that sanguinarine is a promising candidate targeting HIF-1α/TGF-β signaling to improve the treatment for HCC patients.

## Introduction

Hepatocellular carcinoma (HCC), the primary liver malignancy, is the fourth leading cause of cancer-related death, and the incidence rate of HCC has substantially increased worldwide^1^. Despite advances in the diagnosis and treatment of HCC, the 5-year survival rate remains only 18%, the second lowest among all cancer^2,3^. In HCC progression, epithelial–mesenchymal transition (EMT) plays an important role in early steps of metastasis when cells lose contacts and acquire motility to spread into surrounding tissues or metastasize to extrahepatic sites^4^.

Although EMT is a multistep process with complex mechanisms, tumor microenvironment features, including hypoxia and excessive production of transforming growth factor-β (TGF-β), undoubtedly contribute to the induction of EMT^5^. HCC is one of the most hypoxic tumors with median oxygen level as low as 0.8%^6^. As one of the major mediators of hypoxic response, hypoxia inducible factor-1 (HIF-1) senses intratumoral oxygen tension and subsequently mediates activation of hypoxia responses involved in cancer progression, including proliferation, metabolism, angiogenesis, invasion, metastasis and therapy resistance, thus representing a potential anti-HCC target. Under normoxia, oxygen-sensitive HIF-1α is hydroxylated by prolyl hydroxylase domain-containing proteins (PHDs), thereby ubiquitinated and degraded by an E3 ligase, the von Hippel-Lindau tumor suppressor protein. Under hypoxia, stabilized HIF-1α heterodimerizes with constitutively expressed HIF-1β, also called aryl hydrocarbon receptor nuclear translocator (ARNT), in the nucleus and binds hypoxia-response element in the promoter region of target genes^7–9^. HIF-1α drives the expression of many EMT markers and modulators, such as E-cadherin, N-cadherin, and transcriptional activation of Snail^10^.

TGF-β, one of the most potent EMT inducers, has been identified as a therapeutic target in advanced HCC^11,12^. TGF-β activates TGF-β type I receptors which phosphorylate specific receptor-regulated Smad proteins, Smad2 and Smad3, which assemble into heteromeric complexes with Smad4. Heteromeric Smad complexes translocate into the nucleus, where they regulate the transcription of target genes^13^. In addition, Smad-independent signaling pathways, such as PI3K-AKT and MAP kinase pathways, have been implicated in TGF-β-induced EMT non-Smad pathways in TGF-β signaling^14^.

Recently, a few studies have demonstrated the crosstalk between HIF-1α and TGF-β in tumor progression^15,16^. Independent studies have shown that HIF-1α and TGF-β are able to induce EMT, however, the interaction between HIF-1α and TGF-β feed-forward and its contribution to EMT in HCC still await further investigation. We hypothesized the formation of HIF-1α/TGF-β feed-forward loop might contribute to the induction and development of EMT in HCC cells. Numerous attempts have been made to block EMT process by targeting EMT markers^17,18^, EMT-activating transcription factors^19^ or extracelluar inducers^20^. However, regarding the clinical aspect, ongoing trails provided no clinical drugs targeting EMT^21^.

Sanguinarine is a benzophenanthridine alkaloid predominantly isolated from the root of *Sanguinaria canadensis*, *Chelidonium majus* and other medicinal poppy *Fumaria* species. The anticancer potential of sanguinarine has been demonstrated in in vivo and in vitro preclinical studies, including apoptosis inducing, antiproliferative, antiangiogenic, and anti-invasive properties in skin, prostate, cervical, breast, hematological, gastrointestinal, pancreatic, and lung malignancies^22,23^. However, its effects on HIF-1α signaling and TGF-β-mediated EMT in HCC are still unknown.

This study aims to investigate the formation of HIF-1α/TGF-β feed-forward loop that can contribute to the induction and development of EMT in HCC cells. Further, we establish hypoxia and TGF-β-induced EMT models in HCC cells based on the assessment of EMT extent in different cell lines, and evaluate the antiproliferative and EMT reversing effects of sanguinarine in vitro and in vivo. Our study indicates the potential of sanguinarine in HCC treatment and might bring insights to the application of sanguinarine for research and clinical purposes.

## Results

### HIF-1α/TGF-β feed-forward signaling in HCC cells

**Fig. 1:**
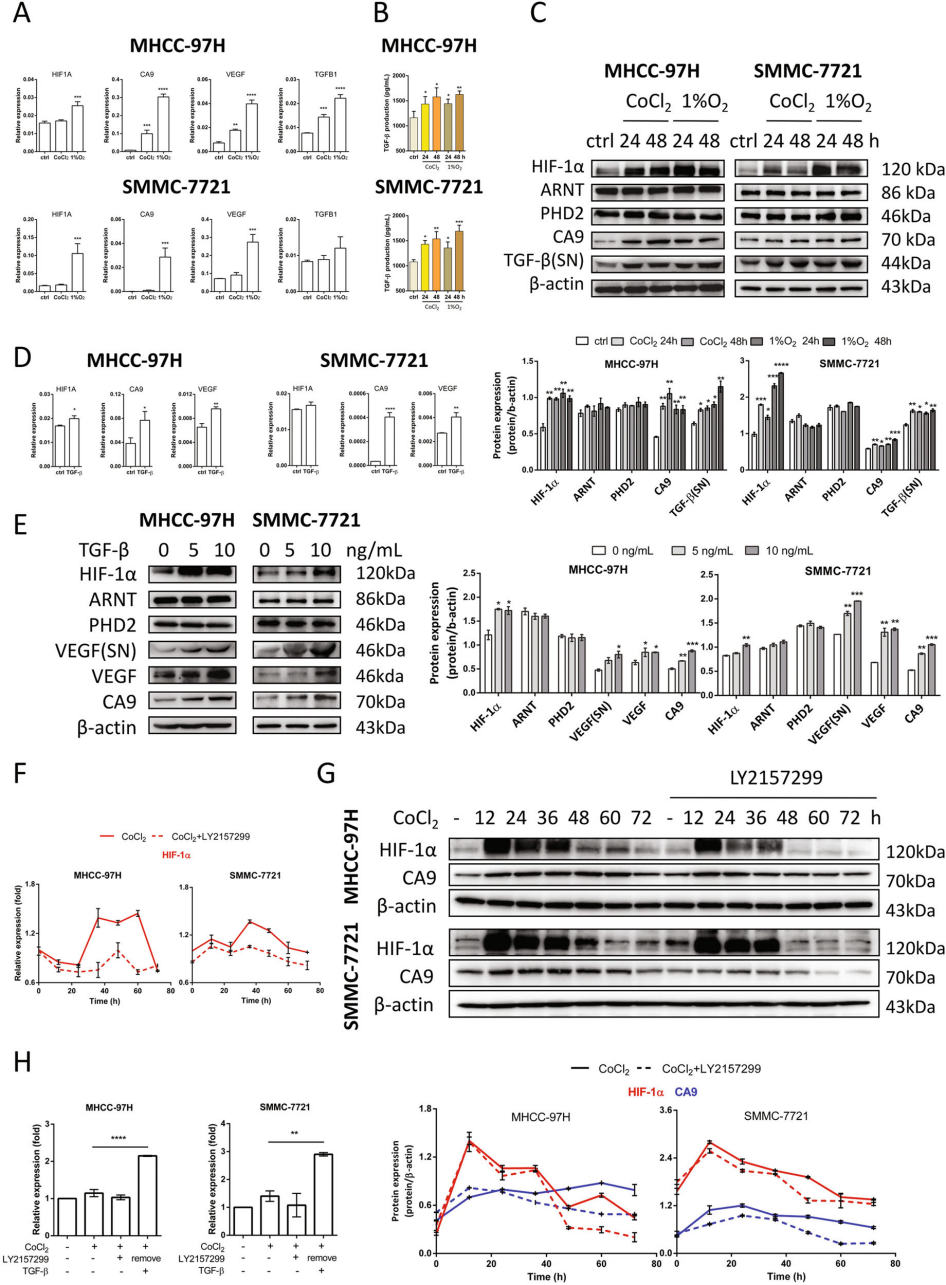
HIF-1α/TGF-β feed-forward loop formation. **a** MHCC-97H and SMMC-7721 cells were treated with 100 μM CoCl_2_ or incubated in 1% O_2_ for 24 h. HIF1A, CA9, VEGF, and TGFB1 mRNA expression values were assessed by RT-qPCR. Gene expression is normalized to ACTB. MHCC-97H and SMMC-7721 cells were treated with 100 μM CoCl_2_ or incubated in 1% O_2_ for 24 or 48 h. **b** TGF-β secretion was determined by ELISA. Mean + SEM (*n* = 5), *P *<* 0.05, **P *<* 0.01, ***P *<* 0.001, ****P *<* 0.0001, one-way ANOVA followed by Bonferroni posttest in comparison with control. **c** HIF-1α, ARNT, PHD2, CA9, and TGF-β (SN, supernatant) protein levels were determined by western blotting. Quantification plots are shown below. **d** MHCC-97H and SMMC-7721 cells were stimulate**d** with 10 ng/mL TGF-β for 24 h. HIF1A, CA9, and VEGF mRNA expression values were assessed by RT-qPCR. Gene expression is normalized to ACTB. **e** MHCC-97H and SMMC-7721 cells were stimulat**e**d with 0, 5, and 10 ng/mL TGF-β for 24 h. HIF-1α, ARNT, PHD2 and VEGF, and CA9 protein levels were examined by western blotting. Quantification plots are shown on the right. **f** MHCC-97H and SMMC-7721 cells were treated with 100 μM CoCl_2_ in the presence of absence of 0.5 μM TGF-β receptor inhibitor LY2157299. HIF1A expression was assessed by RT-qPCR. Gene expression is normalized to ACTB and control group was assigned as 1. **g** MHCC-97H and SMMC-7721 cells were treated with 100 μM CoCl_2_ in the presence of absence of 0.5 μM TGF-β receptor inhibitor LY2157299. HIF-1α and CA9 were determined by western blotting. Quantification plots are shown below. **h** MHCC-97H and SMMC-7721 cells were treated with 100 μM CoCl_2_ in the presence of absence of 0.5 μM LY2157_2_99 for 48 h. LY2157299 was removed and treated with 5 ng/mL TGF-β for additional 24 h. HIF1A expression was assessed by RT-qPCR. Gene expression is normalized to ACTB and shown as fold change. Data were expressed as mean ± SEM (*n* = 3). *P < 0.05, **P < 0.01, ***P < 0.001, ****P < 0.0001 compared with untreated control cells or as indicated. Data are representative of three independent experiments.

To test whether hypoxia affects the TGF-β expression, MHCC-97H and SMMC-7721 cells were cultured with 100 μM CoCl_2_ or under hypoxic conditions (1% O_2_) for 24 h. mRNA and protein levels of HIF-1α, HIF-1α target genes carbonic anhydrase 9 (CA9) and vascular endothelial growth factor (VEGF), as well as TGFB1 were assessed by RT-qPCR and western blotting. 1% O_2_ incubation increased HIF1A expression while CoCl_2_ had little influence on HIF1A gene levels. Under both conditions, enhanced HIF-1α protein levels were observed indicating CoCl_2_ and 1% O_2_ inhibited HIF-1α degradation and 1% O_2_ could also promote HIF-1α gene expression. Activated HIF-1α signaling demonstrated by enhanced CA9 and VEGF gene expression were observed in HCC cell lines (Fig. 1a, c). Importantly, TGF-β gene and protein expression were elevated without alteration of HIF-1α heterodimer partner, ARNT, and HIF-1α hydroxylase, PHD2 protein levels under hypoxia in HCC cells (Figs. 1b, c and S1a), suggesting hypoxia promoted TGF-β signaling. When MHCC-97H and SMMC-7721 cells were treated with 10 ng/mL human recombinant TGF-β for 24 h and HIF1A, HIF-1α target genes CA9 and VEGF gene expression levels were increased (Fig. 1d). Western blot analysis revealed that TGF-β could enhance HIF-1α and targeted protein VEGF levels in both cell lysate and supernatant (SN) (Figs. 1e and S1c). Since HIF-1α induces TGF-β which may further induce HIF-1α, we used CoCl_2_-induced hypoxia models to demonstrate HIF-1α/TGF-β feed-forward signaling in HCC cells. In Fig. 1f, increased HIF1A gene expression was observed after 36 h and blocked in the presence of the TGF-β receptor inhibitor LY2157299 (Galunisertib). In Fig. 1g, HIF-1α activation (CA9 protein levels) through TGF-β was not present compared with control with longer kinetics. When LY2157299 was removed, exogenous TGF-β was added to mimic endogenous secretion, and increased HIF-1α expression (Fig. 1h). Taken together, the data suggested that upregulated HIF-1α expression in hypoxic HCC cells induces TGF-β which further induces and activates HIF-1α to form the HIF-1α/TGF-β feed-forward loop.

### Overexpression of HIF-1α and TGF-β correlated with poor prognosis in HCC

**Fig. 2:**
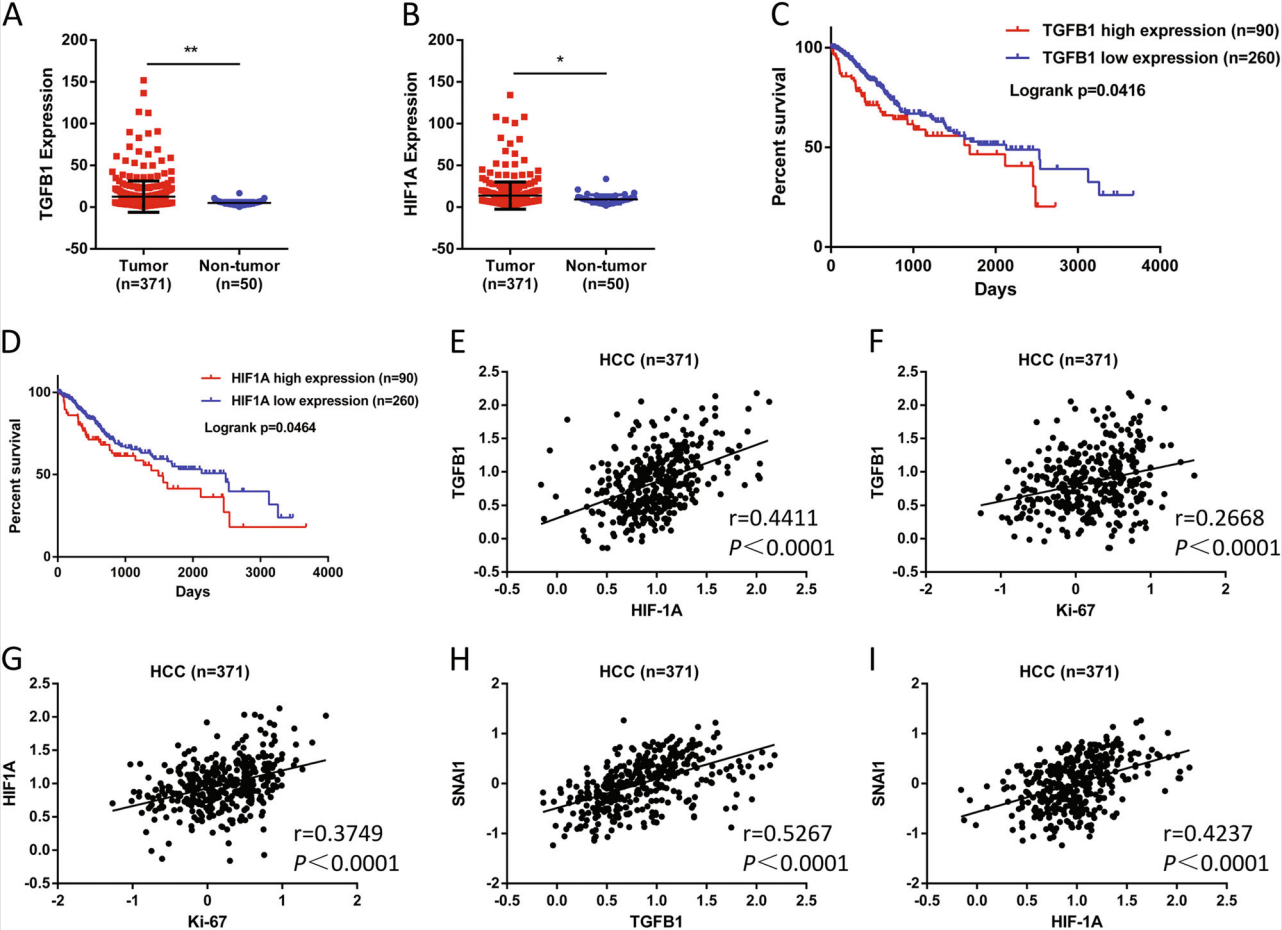
HIF-1α and TGF-β correlated with poor prognosis in HCC. **a** TGFB1 and **b** HIF1A expression in HCC and normal liver tissues based on TCGA dataset (*P *<* 0.05, **P *<* 0.01). Data are represented as mean + SEM. Kaplan–Meier estimates of survival of patients with HCC according to the **c** TGFB1 or **d** HIF1A gene expression. Overall survival curve of the patients with high and low expression of TGFB1. The *P* value obtained from log-rank test. The positive correlation between the expression of **e** TGFB1 and HIF1A, **f** TGFB1 and proliferation marker Ki-67, **g** HIF1A and Ki-67, **h** SNAI1 and TGFB1, **i** SNAI1 and HIF1A.

To confirm the high expression of HIF-1α and TGF-β in vivo, we analyzed data of The Cancer Genome Atlas (TCGA) HCC cohorts and found that TGFB1 and HIF1A expression were significantly overexpressed in 371 tumor specimens compared with 50 non-tumor tissues (Fig. 2a, b). High TGFB1 and HIF1A expression were associated with unfavorable survival outcomes in HCC patients (Fig. 2c, d). Furthermore, as shown in Fig. 2e–g, positive correlations were observed between TGFB1 and HIF1A (*r* = 0.4411, *P* < 0.0001), HIF1A and proliferation marker Ki-67 (*r* = 0.3749, *P* < 0.0001), or TGFB1 and Ki-67 (*r* = 0.2688, *P* < 0.0001). Moreover, mRNA levels of TGFB1 (*r* = 0.5267, *P* < 0.0001) and HIF1A (*r* = 0.4237, *P* *<* 0.0001) were also positively correlated with the EMT marker SNAI1 (Fig. 2h, i).

### Sanguinarine inhibited the proliferation of epithelial and mesenchymal HCC cells

**Fig. 3:**
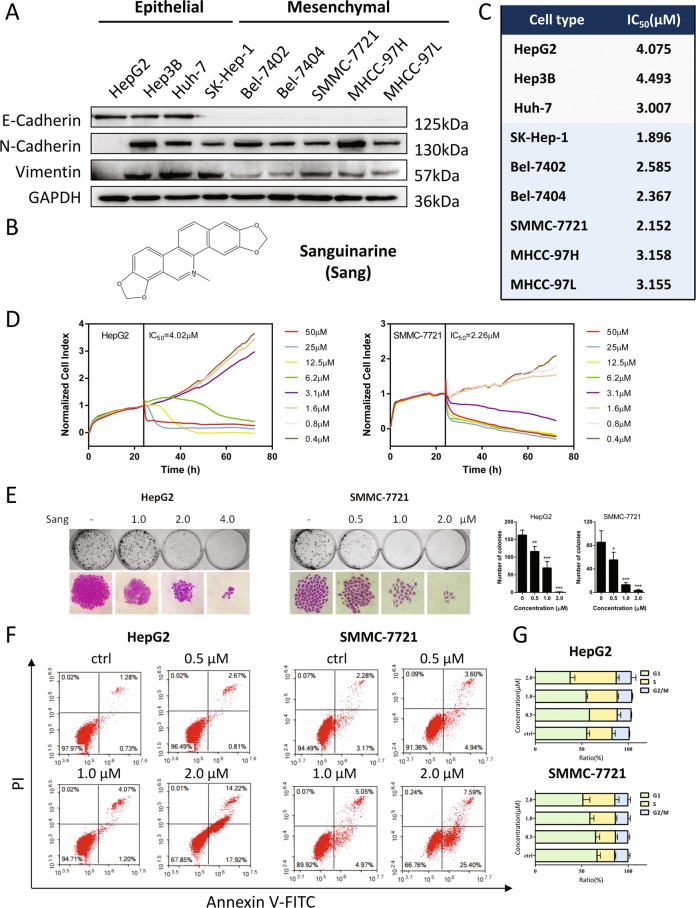
Sanguinarine inhibited HCC cell proliferation. **a** Western blot analysis of E-cadherin, N-cadherin, and Vimentin in nine HCC cell lines: HepG2, Hep3B, Huh-7, SK-Hep-1, Bel-7402, Bel-7404, SMMC-7721, MHCC-97H, and MHCC-97L. **b** Chemical structure of sanguinarine (Sang). **c** HCC cells were treated with for 48 h and cell viability was determined by MTT assay and IC_50_ values were calculated. **d** Real-time cell growth curve of HepG2 and SMMC-7721 cells treated with indicated concentrations of sanguinarine for 48 h. The results shown were representative of three independent experiments. **e** Left: Effect of sanguinarine on the colony formation of HepG2 and SMMC-7721 cells. The colony formation (the upper row) and the individual colony (the lower row) (×200 magnification) were photographed. Right: Quantification of the data presented on the left. *P < 0.05, **P < 0.01, ***P < 0.001, compared with untreated control cells. **f** Cell apoptosis was measured by Annexin V-FITC/PI staining in HepG2 and SMMC-7721 cells treated with sanguinarine. The percentage of apoptotic cells is indicated. **g** Effect of sanguinarine on HepG2 and SMMC-7721 cell cycle progression. Cells were treated with sanguinarine for 48 h and stained with propidium iodide (PI). DNA content was then measured by flow cytometry. Data are represented as mean + SEM.

To determine the EMT extent in HCC cell lines, the expression of E-cadherin, N-cadherin, and Vimentin were analyzed by western blotting (Fig. 3a). While HepG2, Hep3B and Huh-7 were considered to be epithelial based on their expression of E-cadherin, the other six kinds of cell lines (SK-Hep-1, Bel-7402, Bel-7404, SMMC-7721, MHCC-97H, and MHCC-97L) were classified as mesenchymal due to low E-cadherin and high N-cadherin expression, although Vimentin expression varies among the tested cell lines. The effect of sanguinarine (Fig. 3b) on the proliferation of epithelial and mesenchymal HCC cells was analyzed by a MTT assay (Figs. S2 and 3c). The following experiments were performed in HepG2 and SMMC-7721 cells and real-time cell analysis (RTCA) results confirmed the IC_50_ of sanguinarine in both cell lines (Fig. 3c, d). A colony formation assay was also conducted, showing a significant suppression of Hep2G and SMMC-7721 cells by sanguinarine (Fig. 3e). Moreover, Annexin V/PI staining and cell cycle analysis demonstrated that sanguinarine induced apoptosis and cell cycle arrest at S phase in HepG2 and SMMC-7721 cells (Fig. 3f, g).

### Sanguinarine inhibited HCC cell growth in vivo

**Fig. 4:**
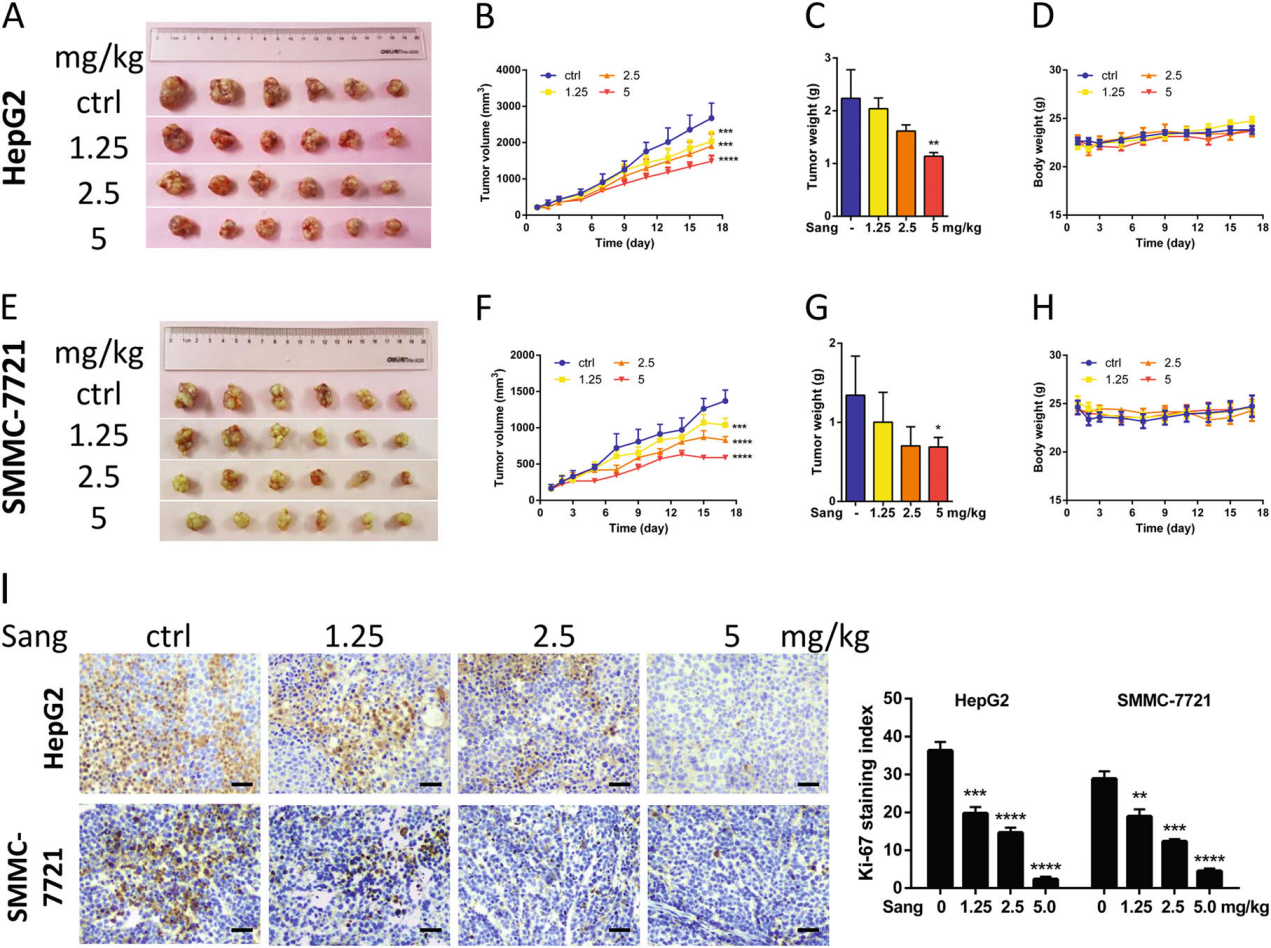
Sanguinarine inhibited the growth of HCC cell xenografts in nude mice. Photograph of tumors from the control group and sanguinarine-treated groups in **a** HepG2 cell and **e** SMMC-7721 cell xenograft models. Tumor volume was measured during the experiment in **b** HepG2 cell and **f** SMMC-7721 cell xenograft models. Tumors were weighed at the end of the experiment in **c** HepG2 cell and **g** SMMC-7721 cell xenografts. Mouse body weight was measured during the experiment in **d** HepG2 cell and **h** SMMC-7721 cell xenograft models. **i** Immunohistochemical (IHC) staining of Ki-67 (left). ×200 magnification Ki-67 staining index was assessed as the percentage of total cells that were Ki-67 positive from six randomly selected high-power fields in xenografts from three mice of each group (right). Statistic analysis were performed by one-way ANOVA followed by Bonferroni posttest, and data are represented as mean + SEM, *n* = 6. *P *<* 0.05, **P *<* 0.01, ***P *<* 0.001, ****P *<* 0.0001 vs. control group.

We tested the ability of sanguinarine to inhibit xenografts from HepG2 and SMMC-7721 cell lines in nude mice models. As shown in Fig. 4a–h, sanguinarine significantly inhibited the tumor growth by decreasing the volume and weight without influencing on the body and organ weight of the mice (Fig. S3a, b). Moreover, Ki-67 staining in both models indicated that sanguinarine reduced the proliferation of HCC cells (Fig. 4i, left). In both tumor models, the Ki-67 staining indexes in sanguinarine-treated groups were significantly lower than those of the tumors in the control groups (Fig. 4i, right).

### Hypoxia and TGF-β induced Smad and PI3K-AKT pathways and EMT in HCC cells

**Fig. 5:**
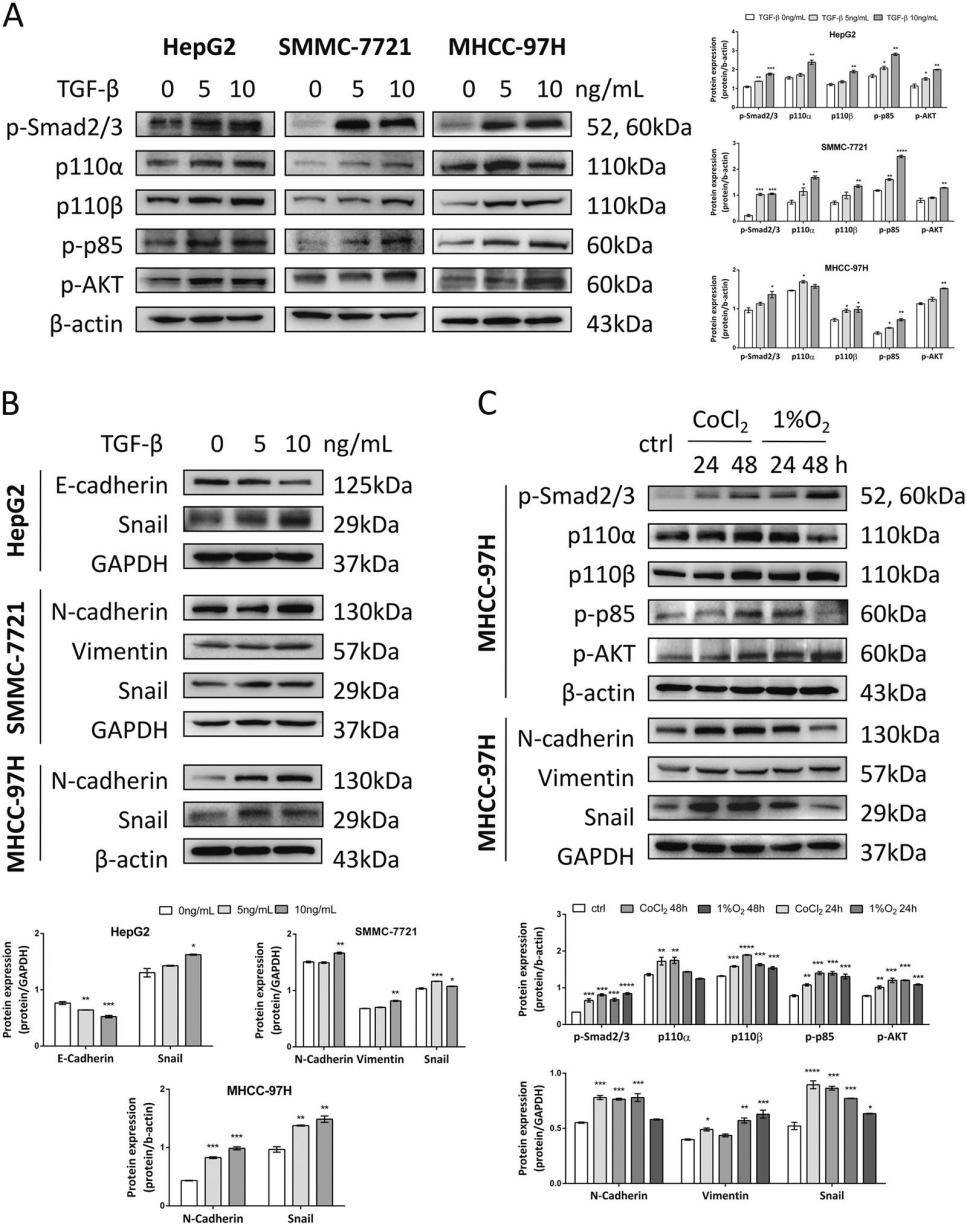
Hypoxia and TGF-β induced EMT via Smad and PI3K-AKT pathways in HCC cells. **a** Western blot analysis of p-Smad2/3, p100α, p110β, p-p85, and p-AKT expression were shown in 0, 5, and 10 ng/mL TGF-β-stimulated HepG2, SMMC-7721, and MHCC-97H cells. Quantification plots are shown on the right. **b** E-cadherin, N-cadherin, Snail and Vimentin expression levels were assessed by western blotting in TGF-β-stimulated HepG2, SMMC-7721 and MHCC-97H cells. Quantification plots are shown below. **c** MHCC-97H cells treated with 100 μM CoCl_2_ or cultured in 1% O_2_ for indicated time. Protein expression of p-Smad2/3, p100α, p110β, p-p85, p-AKT, N-cadherin, Snail, and Vimentin was analyzed by western blotting. GAPDH and β-actin served as controls. Quantification plots are shown below. Data were expressed as mean ± SEM (*n* = 3). *P < 0.05, **P < 0.01, ***P < 0.001, ****P < 0.0001 compared with untreated control cells. The results shown were representative of three independent experiments.

HepG2, SMMC-7721 and MHCC-97H cells were treated with 0, 5, and 10 ng/mL TGF-β for 24 h and the expression levels of proteins involved in canonical Smad, non-canonical PI3K-AKT pathways and EMT were assessed. TGF-β1 activated p-Smad2/3, PI3K-p110α, p110β, p-p85, and p-AKT thus revealing the involvement and activation of Smad and PI3K-AKT pathways (Fig. 5a). Accordingly, CoCl_2_ treatment and 1% O_2_ incubation also activated both pathways (Fig. 5c), which further supported the formation of the HIF-1α/TGF-β feed-forward loop. To test whether EMT is induced by TGF-β and hypoxia, E-cadherin and upregulated N-cadherin, Vimentin and Snail levels suggested that TGF-β, CoCl_2_ or 1% O_2_ treatment induced EMT in HCC cells (Fig. 5b, c).

### Sanguinarine blocked HIF-1α translocation and its signaling

**Fig. 6:**
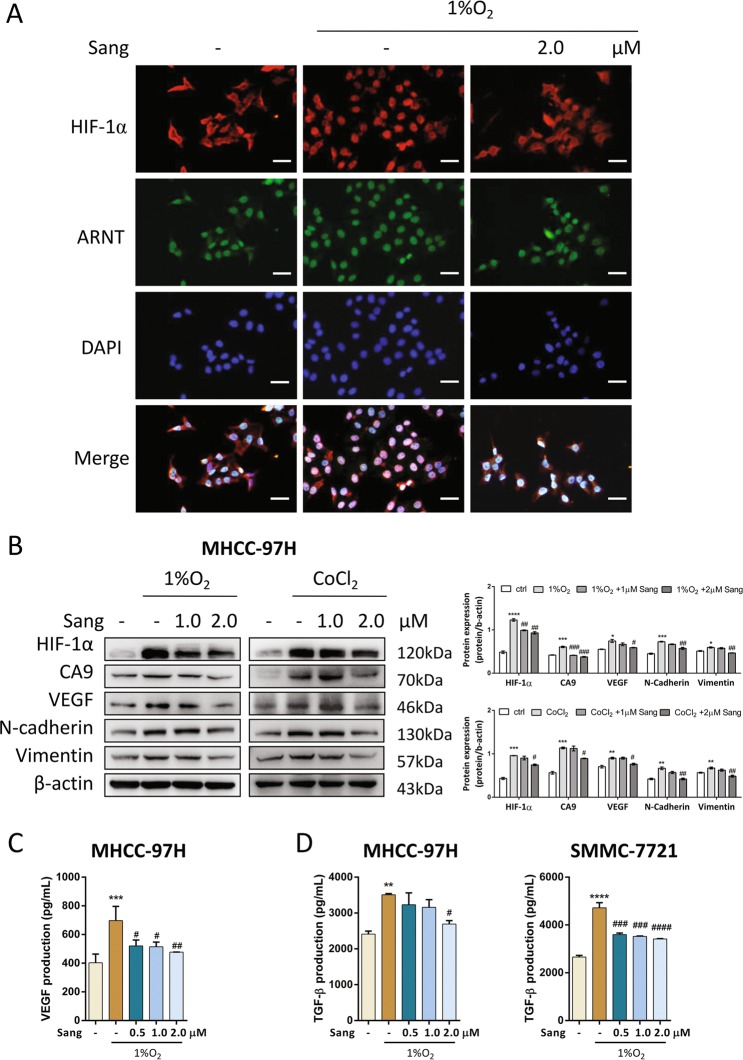
Sanguinarine blocked HIF-1α-induced signaling pathways in vitro. **a** MHCC-97H cells were treated with sanguinarine in the absence or presence of 1% O_2_ for 12 h. HIF-1α (red), ARNT (green), DAPI (blue) staining, and 3-channel merged images indicated the nuclear translocation of HIF-1α. Scale bars, 200 μm. MHCC-97H and SMMC-7721 cells were treated with different concentrations of sanguinarine in the absence or presence of 1% O_2_ or 100 μM CoCl_2_ for 24 h. **b** HIF-1α, CA9, VEGF, N-cadherin, and Vimentin expression levels were assessed by western blotting. Quantification plots are shown on the right. Data were expressed as mean ± SEM (*n* = 3). The results shown were representative of three independent experiments. **c** VEGF and **d** TGF-β production was analyzed by ELISA. Data are represented as mean + SEM. *P *<* 0.05, **P *<* 0.01, ***P < 0.001, ****P < 0.0001, one-way ANOVA followed by Bonferroni posttest in comparison with control. ^*#*^*P* < 0.05, ^*##*^*P* < 0.01, ^###^*P* < 0.001, ^*####*^*P* < 0.0001 one-way ANOVA followed by Bonferroni posttest in comparison with CoCl_2_ or 1% O_2_ samples.

In MHCC-97H cells, 1% O_2_ incubation caused HIF-1α translocation to the nucleus where it heterodimerized with constitutively expressed ARNT initiating downstream signaling. Sanguinarine could effectively inhibited the translocation of HIF-1α as shown in Fig. 6a. We next assessed the effect of sanguinarine on HIF-1α signaling. In cells cultured with CoCl_2_ or 1% O_2_, sanguinarine not only inhibited HIF-1α protein levels and HIF-1α targeted proteins, CA9 and VEGF, but also reduced the protein expression of hypoxia-increased EMT markers, N-cadherin, Vimentin and Snail (Fig. 6b, c). Subsequently, we found that sanguinarine inhibited hypoxia-induced TGF-β secretion, suggesting that sanguinarine affected HIF-1α/TGF-β signaling (Fig. 6d).

### Sanguinarine blocked TGF-β-induced EMT through inhibiting both Smad and PI3K-AKT pathways in vitro

**Fig. 7:**
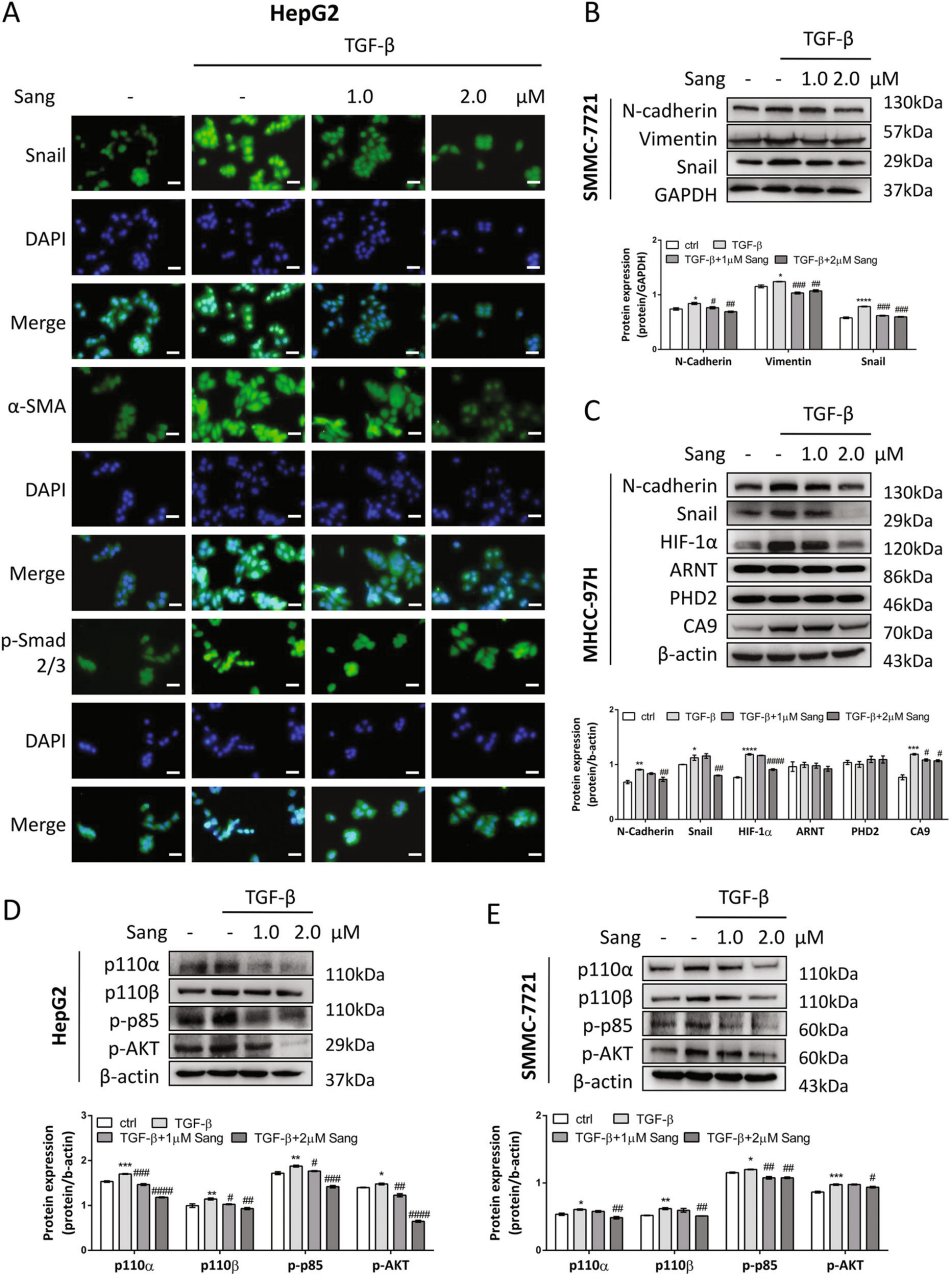
Sanguinarine inhibited TGF-β-induced EMT in vitro. **a** HepG2 cells were treated with different concentrations of sanguinarine in the absence or presence of 10 ng/mL TGF-β for 24 h. Snail (green), α-SMA (green), p-Smad2/3 (green), DAPI (blue) staining and 2-channel merged images indicated the nuclear translocation of Snail and p-Smad2/3, and expression of α-SMA. The results shown were representative of three independent experiments. Scale bars, 200 μm. **b** SMMC-7721 cells were treated with different concentrations of sanguinarine in the absence or presence of 10 ng/mL TGF-β. Protein levels of N-cadherin, Vimentin, and Snail were analyzed by western blotting. Quantification plots are shown below. **c** MHCC-97H cells were treated with different concentrations of sanguinarine in the absence or presence of 10 ng/mL TGF-β. Protein levels of N-cadherin, Snail, HIF-1α, ARNT, PHD2, and CA9 were analyzed by western blotting. GAPDH or β-actin served as controls. Quantification plots are shown below. Western blot analysis of p100α, p110β, p-p85, and p-AKT expression were demonstrated in **d** HepG2 and **e** SMMC-7721 cells treated with indicated concentrations of sanguinarine in the absence or presence of 10 ng/mL TGF-β for 48 h. Quantification plots are shown below. Data were expressed as mean ± SEM (*n* = 3). *P < 0.05, **P < 0.01, ***P < 0.001, ****P < 0.0001 compared with untreated control cells. The results shown were representative of three independent experiments. ^*#*^*P* *<* 0.05, ^*##*^*P* < 0.01, ^*###*^*P* < 0.001, ^*####*^*P* < 0.0001 one-way ANOVA followed by Bonferroni posttest in comparison with TGF-β-treated samples.

To investigate whether sanguinarine could inhibit TGF-β-induced EMT, nuclear translocation of EMT transcription factor, Snail, as well as the expression of α-SMA were evaluated by immunofluorescence staining. The results indicated that sanguinarine decreased Snail translocation and expression, as well as α-smooth muscle actin (α-SMA) expression induced by TGF-β in HepG2 cells (Figs. 7a, and S4a). In basal conditions, SMMC-7721 and MHCC-97H cells exert certain mesenchymal characteristics which could be downregulated by sanguinarine (Fig. S4b–e). Moreover, sanguinarine suppressed TGF-β-induced N-cadherin, Vimentin, and Snail expression in SMMC-7721 cells (Fig. 7b). Beside the inhibition of EMT markers, we found sanguinarine could block TGF-β-enhanced HIF-1α and CA9 expression without changing the level of ARNT and PHD2, indicating the inhibition of the TGF-β/HIF-1α feed-forward loop by sanguinarine (Fig. 7c).

To gain insights to the underlying mechanism, Smad and PI3K-AKT signaling pathway proteins were examined. The results showed that sanguinarine blocked p-Smad2/3 translocation to the nucleus (Fig. 7a) and the activation of PI3K catalytic subunits, p110α and p110β, regulatory subunit p85, as well as AKT in both HepG2 and SMMC-7721 cell lines (Fig. 7d, e).

EMT allows tumor cells to acquire migration properties. Therefore, we tested whether sanguinarine could inhibit TGF-β-induced cell migration in a scratch assay and transwell migration assay. TGF-β-induced scratch closure was significantly inhibited in the presence of sanguinarine in HepG2 and SMMC-7721 cells (Fig. S5a). Furthermore, the transwell assay demonstrated that sanguinarine prevented both cell lines from migrating to the lower surface of the chamber (Fig. S5b).

### Sanguinarine inhibited TGF-β-induced HCC cell EMT in vivo

**Fig. 8:**
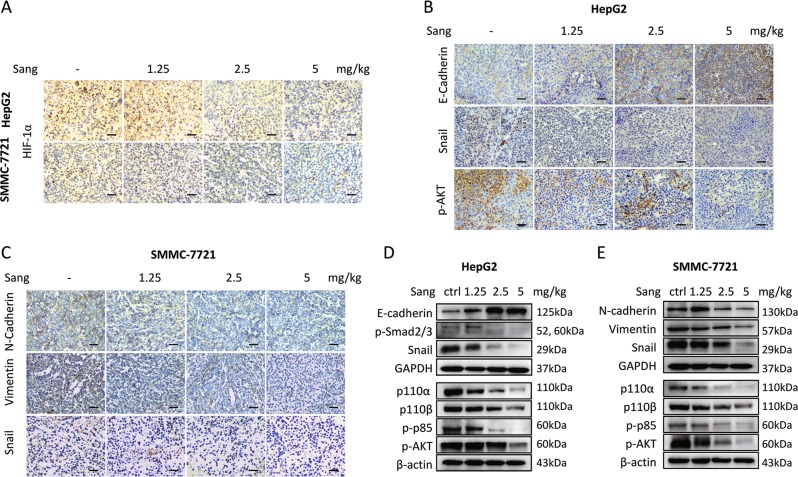
Sanguinarine blocked TGF-β-induced HCC cell EMT in vivo. **a** Tumor sections from xenograft models were immunostained with anti-HIF-1α antibody. **b** IHC staining of E-cadherin, Snail and p-AKT in HepG2 xenografts. **c** IHC staining of N-cadherin, Vimentin and Snail in SMMC-7721 xenografts. ×200 magnification. **d** Western blot analysis of E-cadherin, p-Smad2/3, Snail, p110α, p110β, p-p85, and p-AKT expression in HepG2 xenograft tumor samples. magnification **e** Western blot analysis of N-cadherin, Vimentin, Snail, p100α, p110β, p-p85, and p-AKT protein levels in SMMC-7721 xenograft tumor samples. The results shown were representative of three independent experiments.

In HepG2 and SMMC-7721 xenograft models, immunohistochemistry and western blot analysis of the tumor samples showed that in accordance with in vitro results, sanguinarine could inhibit the expression change of HIF-1α (Fig. 8a), EMT markers, including E-cadherin and Snail in HepG2 (Fig. 8b) so as to N-cadherin, Vimentin, and Snail in SMMC-7721 xenograft models (Fig. 8c). Both Smad and PI3K-AKT pathway proteins were also downregulated in sanguinarine-treated groups (Fig. 8d).

## Discussion

Proliferation and EMT are two major pathological changes in hepatocellular carcinoma (HCC) development. Inhibiting the proliferation and EMT of HCC cells can be reasonable strategies for HCC treatment. Hypoxia and an excessive amount of TGF-β are two central modulators in tumor EMT. In this study, we found that HIF-1α and TGF-β form a positive feed-forward loop to induce HCC cell EMT. Sanguinarine could inhibit HCC cell proliferation, HIF-1α/TGF-β feed-forward loop, EMT and migration in vitro and in vivo.

Accumulating evidence has suggested the crosstalk between hypoxia and TGF-β signaling in cancer cells. On one hand, in line with our observations that hypoxia increases TGF-β expression and signaling in HCC cells, hypoxia triggers extracellular matrix-related protein expression in a TGF-β- and PI3K-dependent manner in pancreatic cancer cells^24^, or induces EMT through TGF-β autocrine signaling in gastric cancer cells^25^. One the other hand, TGF-β-induced HIF-1 stabilization in HepG2 cells^26^ is consistent with our findings that TGF-β enhances HIF-1α expression as well as its targeted CA9 and VEGF transcription. Taken together, our study has suggested the existence of a HIF-1α/TGF-β feed-forward loop in HCC cells and its role in EMT induction. Notably, TCGA data showed higher HIF1A and TGFB1 gene expression in HCC tissues and its correlation with the overall survival rate, tumor cell proliferation and EMT. Thus, targeting the HIF-1α/TGF-β feed-forward loop could be a feasible therapeutic strategy for HCC patients.

Sanguinarine has potent antiproliferative effects in nine kinds of tested HCC cell lines. Sanguinarine also inhibited the colony formation of HepG2 and SMMC-7721 cells. Furthermore, reduced xenograft tumor volume and weight and Ki-67 expression confirmed that sanguinarine could inhibit the proliferation of HepG2 and SMMC-7721 cells in vivo.

The role of EMT in tumor progression has been intensively studied in the past decade. E-cadherin downregulation and N-cadherin upregulation lead to an increase in cellular motility. Loss of E-cadherin or increase of N-cadherin expression has been implicated in cancer progression and metastasis^27,28^. As a major cytoskeletal component of mesenchymal cells, Vimentin is another marker often used for mesenchymal-derived cells or cells undergoing EMT during metastatic progression^29^. Besides, targeting EMT has been considered as a potential way to overcome drug resistance in cancer treatment^30^. Characterizing hepatoma cell lines as epithelial or mesenchymal by E/N-cadherin and Vimentin expression facilities us better understanding of the cell EMT extent. Studies have shown a strong correlation between EMT extent and the sensitivity of anticancer drugs^31,32^. We chose HepG2, SMMC-7721 and MHCC-97H cells to conduct the experiments and observed that TGF-β-induced changes of EMT markers in these cell types, including decrease of E-cadherin expression, increase of N-cadherin, Vimentin and α-SMA. Moreover, EMT-activating transcription factors, including Snail, Twist and Zeb families, executing EMT and playing important roles in cancer progression from tumor initiation, growth, invasion, dissemination, and metastasis to colonization as well as in drug resistance^33^. Nuclear translocation of EMT transcription factor Snail and elevated Snail expression were found in TGF-β-treated cells. These findings suggested that TGF-β could induce EMT in epithelial and also mesenchymal cells. In accordance with the knowledge that cells achieved migration ability through EMT, we observed that TGF-β enhanced cell migration in both cell lines.

The prognosis of metastasized HCC is particularly poor due to lack of treatment choices. Higher serum levels of TGF-β in HCC patients were found to be associated with less sensitivity to sorafinib, the first regimen listed in the treatment for advanced HCC^34^. TGF-β signaling should be explored as a therapeutic target to improve treatments for advanced HCC. Indeed, blocking TGF-β has been found to elevate E-cadherin levels and reduce the migration and invasion of HCC cells^35^. Moreover, TGF-β receptor inhibitors are the only compounds interfering with EMT in clinical trial and have shown the ability to inhibit HCC cell migration and invasion^21,36^. This study demonstrated that sanguinarine as a natural compound exerted EMT reversing effects in hypoxia- or TGF-β-treated HCC cells. In vivo studies also confirmed that sanguinarine treatment inhibited the local EMT process which may be the result from the control of both HIF-1α and TGF-β signaling. Although the secretion of TGF-β and VEGF were unaffected by sanguinarine in tumor tissue samples, the involvement of multiple cells in a local area may contribute to the average production. In TGF-β-induced EMT and cell migration models, we demonstrated that sanguinarine could decrease the expression of EMT markers, Snail translocation and cell migration. In addition, sanguinarine also suppressed protein expression of N-cadherin and Vimentin and inhibited SMMC-7721 cell migration without TGF-β stimulation.

Hypoxia and TGF-β activated both Smad and PI3K-AKT pathways in HepG2 and SMMC-7721 cells, indicating these pathways may be the underlying mechanism of induced EMT in HCC cells. To elucidate sanguinarine's mode of action, we found that EMT reversing effects of sanguinarine were resulted from inhibition of these pathways. TGF-β is one of the main drivers of liver fibrosis which is one of the main precancerous conditions for HCC. Inhibition of TGF-β signaling by sanguinarine may also have potential anti-fibrosis effects in liver cells preventing the induction of HCC, while TGF-β seems to have various roles depending on tumor type and stage, e.g., TGF-β inhibits cell proliferation in early stages of many malignancies, but promotes cancer progression or late-stage tumors^37^. Further studies regarding the effects of sanguinarine on liver fibrosis and different stages of HCC are still needed.

In summary, the HIF-1α/TGF-β feed-forward loop could be a target for HCC treatment. Sanguinarine could target HIF-1α and TGF-β signaling pathways and inhibit subsequent EMT and cell migration. Given the properties found in this study, sanguinarine can be a candidate as an adjuvant in cancer therapy for HCC patients.

## Materials and methods

### Chemicals and reagents

Sanguinarine (HPLC ≥ 99%, Lot: HS026156198) was purchased from Baoji Kerui Biochemical Pharmaceutical Co., Ltd (Shaanxi, China). Dulbecco’s modified Eagle’s medium (DMEM), 3-(4, 5-Dimethylthiazol-2-yl)-2.5-diphenyl-2H-tetrazolium bromide (MTT), trypsin, and dimethyl sulfoxide (DMSO) were purchased from Sigma-Aldrich (St. Louis, MO, USA). Penicillin and streptomycin were purchased from Harbin General Pharmaceutical Factory (Harbin, China) and North China Pharmaceutical (Shijiazhuang, China), respectively. Fetal bovine serum (FBS) was purchased from Excell Bio (Shanghai, China). Crystal violet was purchased from Beijing Chemical Plant (Beijing, China). E-cadherin, N-cadherin, Vimentin, Snail, VEGF, ARNT, CA9, PHD2, TGF-β rabbit mAb, Phospho-AKT, HIF-1α, GAPDH, and β-actin mouse mAb were obtained from Protein Technology Group (Chicago, Illinois, USA), and PI3K-p110α, PI3K-p110β, Phospho-p85, Phospho-Smad2/3, and α-SMA rabbit mAb were purchased from Cell Signaling (Boston, Massachusetts, USA). All the antibodies were used in the dilution of 1:1000 for western blotting. RIPA Lysis Buffer was obtained from Applygen Technologies (Beijing, China). Protease inhibitor cocktail and phosphatase inhibitor cocktail were purchased from Roche Technology (Basle, Switzerland). CoraLite488-goat anti-rabbit and CoraLite594-goat anti-mouse IgG, BCA protein assay reagent kit and enhanced chemiluminescent (ECL) plus reagent kit were obtained from Pierce Biotech (Rockford, Illinois, USA). Human recombinant TGF-β were purchased from PeproTech Inc. (Rocky Hill, Connecticut, USA). LY2157299 (Galunisertib) was obtained from TargetMol (Shanghai, China).

### Cell lines and cell culture

Human liver cancer cell line HepG2, Hep3B, Huh-7, SK-Hep-1, Bel-7402, Bel-7404, SMMC-7721, MHCC-97H, and MHCC-97L were obtained from Shanghai Institute of Cell Biology in the Chinese Academy of Sciences. HepG2, Hep3B, and Huh-7 cells were cultured in DMEM medium (4.5 mg/mL glucose, 2 mmol/L L-glutamine and sodium pyruvate) with 10% (v/v) FBS, supplemented with 100 units/mL penicillin and 100 mg/mL streptomycin; Bel-7402, Bel-7404, SMMC-7721, MHCC-97H, and MHCC-97L cells were cultured in RPMI-1640 medium with 10% (v/v) FBS, supplemented with 100 units/mL penicillin and 100 mg/mL streptomycin; SK-Hep-1 cells were cultured in MEM medium with 10% (v/v) FBS, supplemented with 100 units/mL penicillin and 100 mg/mL streptomycin. The cell lines were authenticated by STR profiling and tested for mycoplasma contamination. Cells were maintained at 37 °C in a humidified incubator with 5% CO_2_ in air.

### Cell proliferation assay

HepG2, Hep3B, Huh-7, SK-Hep-1, Bel-7402, Bel-7404, SMMC-7721, MHCC-97H, and MHCC-97L cells were seeded into 96-well plates (1 × 10^4^ cells/well) and cultured with different concentrations of sanguinarine for 48 h. After treatment, cells were cultured with serum-free medium and MTT (0.5 mg/mL) for 4 h. After the removal of medium,150 μL DMSO was added to each well and the plates were shaken for 15 min. The plates were then analyzed using a microplate reader (Bio-Rad, Hercules, CA, USA) using a wavelength of 490 nm. Three independent experiments, each with triplicate cultures, were performed to determine each data point.

### Real-time cell analysis

Real-time cell analysis (RTCA) was conducted using the xCELLigence RTCA S16 (ACEA, San Diego, USA). Briefly, 50 μL of the medium was added to 16-well E-Plates to obtain background readings before adding 100 μL of the cell suspension (4 × 10^4^ cells/well). After incubation at room temperature for 30 min, the E-Plates were placed on the reader in an incubator, and the cell index recording continued. After 24 h, the cells were treated with sanguinarine at different concentrations. Cells were monitored every 15 min for 48 h after treatment.

### Colony-forming assay

Cells were seeded in a 12-well plate (200 cells/well) and treated with different concentrations of sanguinarine for 48 h. The medium was changed to drug-free medium for additional 10–15 days. Then, the colonies were fixed with methanol and stained with 0.2% crystal violet for 15 min at room temperature. Images were taken using the white light imaging system (Champchemi Professional SG2010084, Sage Creation, Beijing, China) and the inverted fluorescence microscope (DM505, Nikon Co., Ltd., Otawara, Tochigi, Japan).

### Flow cytometry for cell cycle and apoptosis analysis

Cells were plated into 6-well plates. After 24 h incubation, cells were treated with the indicated concentrations of sanguinarine for 48 h. Then cells were harvested and subjected to sequential staining with 5 μL AnnexinV-FITC and 10 μL PI (20 μg/mL) was added and incubated for 10 min.

Cells were cultured in 6-well plates and treated with the indicated concentrations of sanguinarine for 48 h after serum starvation for 24 h. Then the cells were harvested, and fixed in 70% ice-cold ethanol at −20 °C overnight. After fixation, the cells were incubated with 1 mL RNase (50 μg/mL) for 30 min and stained with 1 mL PI (60 μg/mL). All stained cells were analyzed by FACS (Becton Dickinson, Mountain View, CA, USA). The obtained data were analyzed with Modfit LT software.

### Scratch assay

Cells were seeded into a 12-well plate and allowed to grow to 80% confluence in complete medium. Cell monolayers were scratched by a 200 μL pipette tips and plates were then washed three times with PBS and incubated in medium with different stimulation for 48 h. Cell migration into the scratched surface was photographed under an inverted fluorescence microscope (×200 magnification).

### Transwell migration assay

The in vitro migration assay was performed using transwell chambers. Cells were incubated with treatment for 48 h. The medium in the chamber was replaced with serum-free medium and 30% FBS contained medium was added to the 24-well plate as the chemoattractant. After incubation for 24 h, all the non-migrated cells which were located on the upper surface of the chamber were carefully removed with a cotton swab. The migrated cells through the chamber to the lower surface were fixed with 4% paraformaldehyde and then stained with crystal violet for 15 min. The number of migrated cells was photographed under an inverted fluorescence microscope (×200 magnification).

### RNA isolation and RT-qPCR

Total RNA isolation, cDNA synthesis and quantitative RT-PCR were performed as described^38^. Primers with the following sequences were used: TGFB1 5′-GCCGACT ACTACGCCAAGGA-3′ and 5′-ATGCTGTGTGTACTCTGCTTGAAC-3′, HIF1A 5′-CTCATCAGTTGCCACTTCCACATA-3′ and 5′-AGCAATTCATCTGTGCTTTC ATGTC-3′, CA9 5′-ACCAGACAGTGATGCTGAGTGCTAA-3′ and 5′-TCAGCTGT AGCCGAGAGTCACC-3′, VEGF 5′-TCACAGGTACAGGGATGAGGACAC-3′ and 5′-CAAAGCACAGCAATGTCCTGAAG-3′, and ACTB 5′-TGGCACCCAGCACAA TGAA-3′ and 5′-CTAAGTCATAGTCCGCCTAGAAGCA-3′. The relative expression of RNA for each gene was normalized and presented as the ratio of the mRNA value of a target gene to that of the ACTB gene.

### Western blotting

After treatment, cells were lysed in RIPA buffer containing phosphatase and protease inhibitors. Total protein content was quantified with the BCA assay. Secreted protein in the cell supernatant was concentrated by methanol-chloroform precipitation. The protein sample was subjected to SDS-PAGE and transferred to a polyvinylidene difluoride membrane (Millipore, Bedford, MA, USA), which was blocked with 5% non-fat milk in TBST buffer and incubated with the indicated primary antibodies at 4 °C overnight. Then the membrane was incubated with species-specific horseradish peroxidase (HRP)-conjugated secondary antibodies and antigen-antibody complexes were visualized using the enhanced ECL kit. The images were taken using Tanon5200 imaging system (Tanon, Shanghai, China). β-actin and GAPDH were used as control for normalizing protein loading.

### Immunofluorescence

Cells were fixed with 4% paraformaldehyde for 10 min and then blocked with 10% BSA for 30 min at room temperature. After this procedure, the cells were incubated with primary antibodies (1:200) at 37 °C for 4 h. Then, cells were incubated with an CoraLite488-conjugated anti-rabbit and CoraLite594-conjugated anti-mouse secondary antibody (1:50) at room temperature for 1 h. The nucleus was stained with DAPI. Fluorescent signals were detected using an inverted fluorescence microscope (DM505, Nikon Co., Ltd., Otawara, Tochigi, Japan).

### ELISA

The cell culture supernatant was assayed for TGF-β and VEGF (Sino Biological, Beijing, China) by using commercially available ELISA kits.

### Animals and xenograft models

All the experiments were in accordance with the guidelines of the Institutional Animal Care and Use Committee (approval number: 2019-1031). For the induction of xenograft model, 200 μL cell suspension (2 × 10^7^ cells/mL) was subcutaneously implanted into the right axillary of the nude mice (male immune-deficient BABL/c nude mice). When the average of the tumor volume reached about 100 mm^3^, mice were randomly divided into control group and treatment groups (*n* = 6 per group). Mice in the control group were orally administered with 0.5% sodium carboxymethyl cellulose (CMC-Na) solution or different concentrations (1.25, 2.5, 5 mg/kg) of sanguinarine. Animals in every group received ten times of drug administration for consecutive 3 days and every other day during which the body weight and tumor volume were monitored and recorded. On the last day, after the mice were sacrificed, tumors and main organs were removed and weighed. The tumor volume was calculated with the following formula: Tumor volume = *A* × *B* × *B*/2, where *A* was the longest and *B* was the shortest dimension of the tumor. Each tumor was cut in half, one was immersed in 0.1% diethyl pyrocarbonate solution and stored at −80 °C for western blot analysis, and the other half was fixed in 4% paraformaldehyde for immunohistochemistry study.

### Immunohistochemistry assay

Tumor specimens were embedded in paraffin and cut into 5-μm sections for IHC. The SV histostain kit (Boster Bioengineering Co., Ltd, Wuhan, China) was used according to the manufacturer's instructions. The antibodies used in IHC were Ki-67 (1:2000), TGF-β (1:100), HIF-1α (1:200), E-cadherin (1:1000), Snail (1:200), p-AKT (1:200), N-cadherin (1:2000), and Vimentin (1:200). As a measure of proliferation, the Ki-67 staining index was determined as the ratio of labeled nuclei: total nuclei in high-power (×400) fields. Approximately 2000 nuclei were counted in each case by systematic random sampling.

### Statistical analysis

One-way analysis of variance (ANOVA) and further Bonferroni’s multiple comparison test was used and *P* value < 0.05 was considered statistically significant (Prism 6.0, GraphPad, La Jolla, CA, USA). Data are presented as mean + SEM.

## Supplementary information

Supplementary Figures and Legends
