# Supplementary material for: Sanguinarine inhibits epithelial–mesenchymal transition via targeting HIF-1α/TGF-β feed-forward loop in hepatocellular carcinoma
Source: Cell Death Dis. 2019 Dec 9;10(12):939. doi: 10.1038/s41419-019-2173-1 (PMC6901539; doi:10.1038/s41419-019-2173-1)
Supplement: Supplementary file 1 — Supplementary Figures and Legends [file 41419_2019_2173_MOESM1_ESM.doc]

**Supplementary Figure Legends**

**
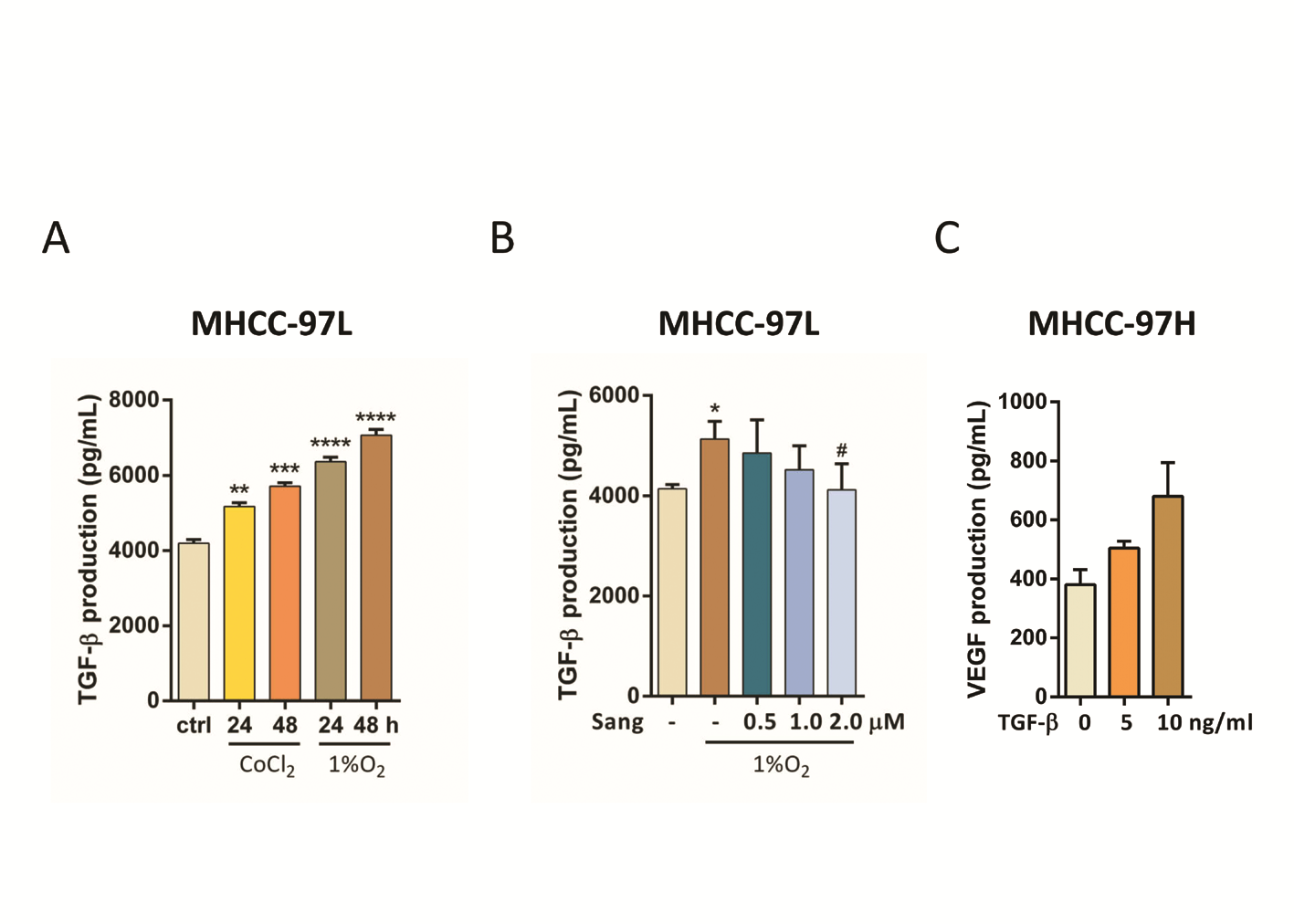
**

**Supplementary Fig. S1.** Sanguinarine inhibited hypoxia-induced TGF-β secretion. **a** MHCC-97L cells were incubated in 100 μM CoCl_2_ or 1% O_2_ for 24 or 48 h. TGF-β secretion was determined by ELISA. **b** MHCC-97L cells were incubated in 1% O_2_ and treated with indicated concentrations of sanguinarine for 24 h. TGF-β secretion was determined by ELISA. **c** MHCC-97H cells were stimulated with TGF-β for 24 h and VEGF production was determined by ELISA. **P < 0.05, **P < 0.01, ***P <0.001,****P < 0.0001*, one-way ANOVA followed by Bonferroni posttest in comparison with control. *#P < 0.05* one-way ANOVA followed by Bonferroni posttest in comparison with 1% O_2_ samples.


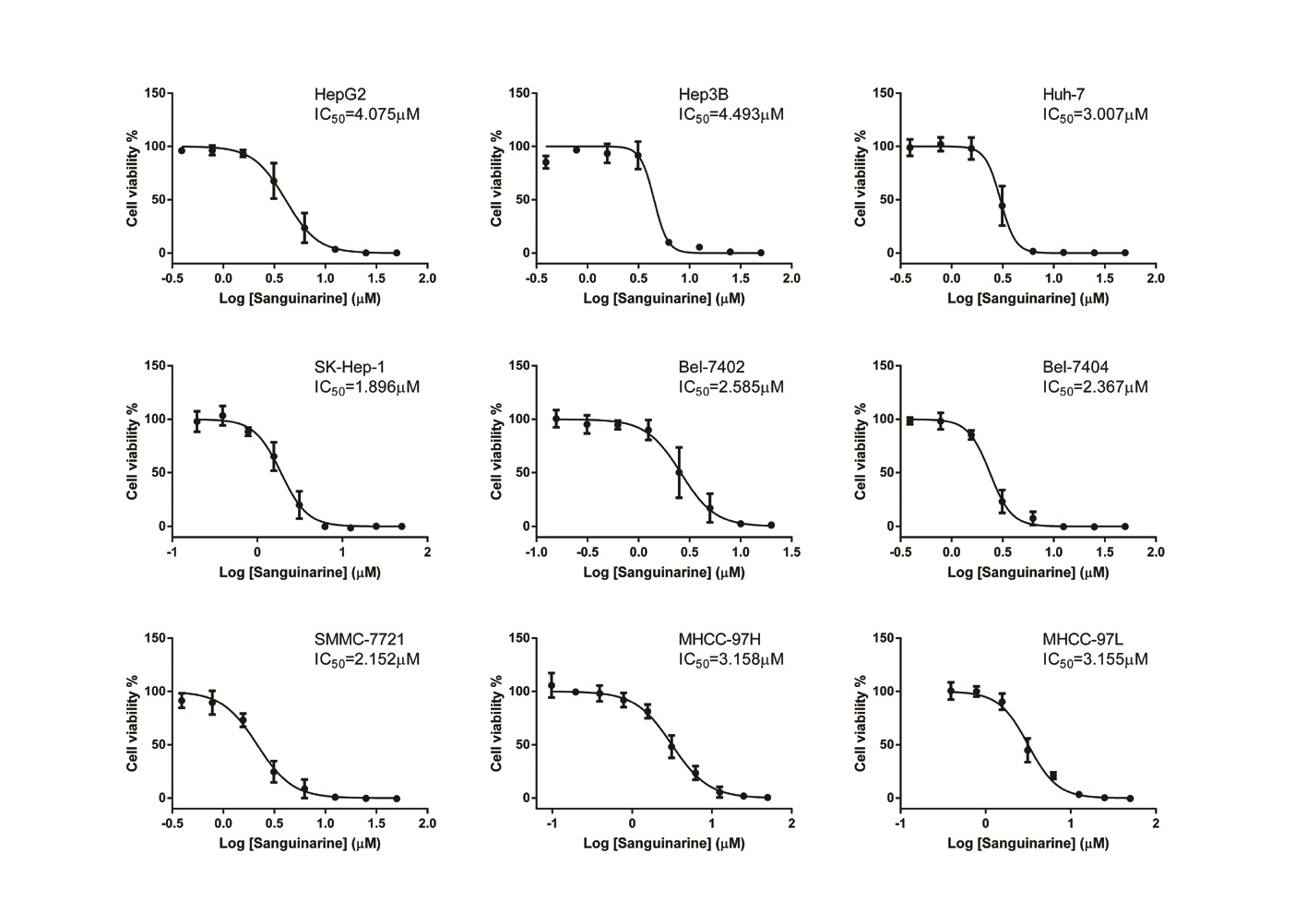


**Supplementary Fig. S2.** Sanguinarine inhibites the proliferation of HCC cells. HepG2, Hep3B, Huh-7, SK-Hep-1, Bel-7402, Bel-7404, SMMC-7721, MHCC-97H and MHCC-97L cells were treated with different concentrations of sanguinarine for 48 h. Cell viability was determined by MTT assay (n=3).


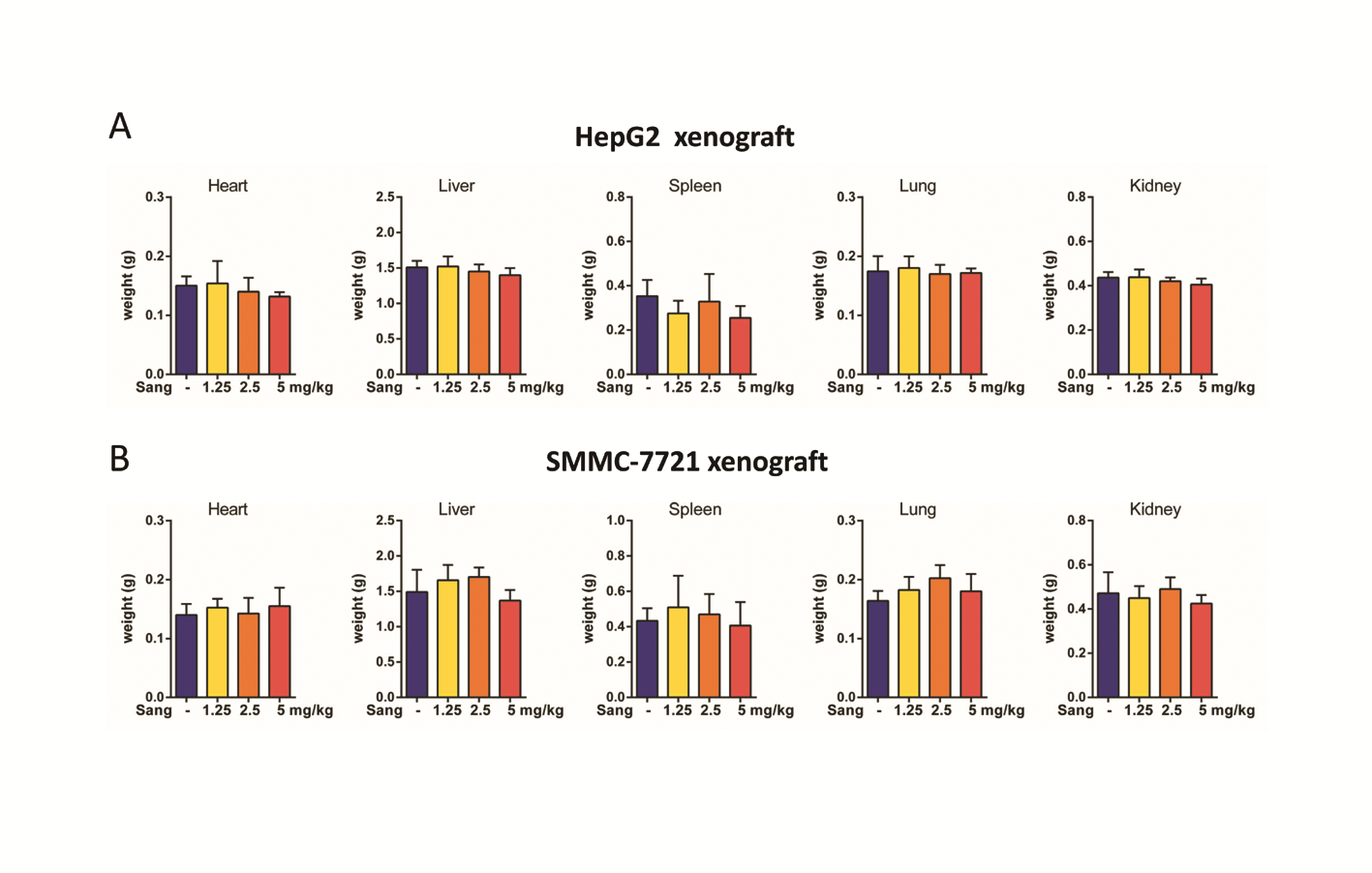


**Supplementary Fig. S3.** Organ weights were unaffected by sanguinarine. **a, b** Hearts, livers, spleens, lungs and kidneys were weighed at the end of the experiment in HepG2 and SMMC-7721 xenografts.


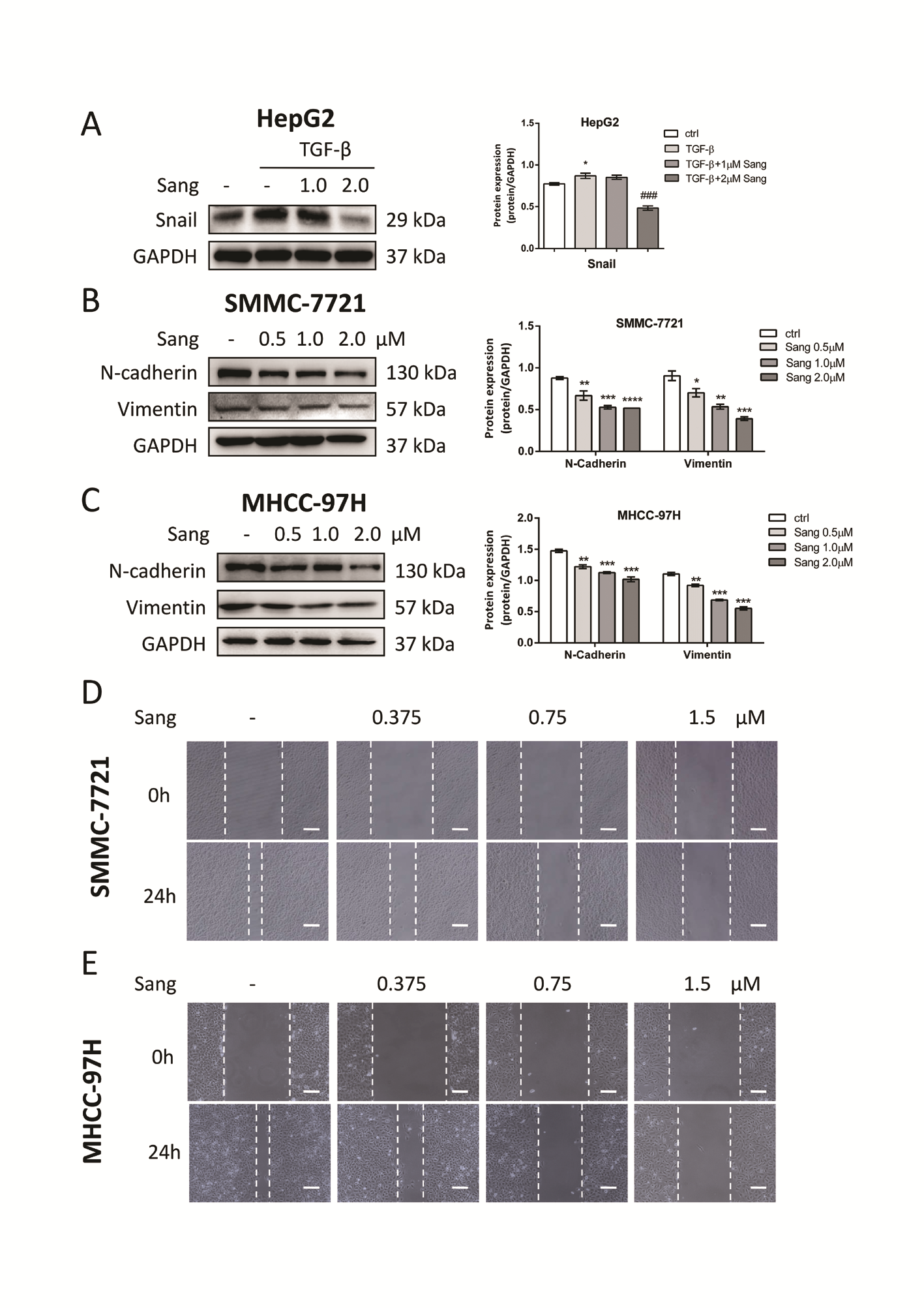


**Supplementary Fig. S4.** Sanguinarine inhibited HCC cell migration and EMT marker expression. **a** HepG2 cells were treated with indicated concentrations of sanguinarine in the absence or presence of 10 ng/mL TGF-β. Protein expression of Snail was measured by western blotting. Quantification plots are show on the right. Data were expressed as mean ± SEM (n = 3). **P* < 0.05, one-way ANOVA followed by Bonferroni posttest in comparison with control. ###*P* < 0.001, one-way ANOVA followed by Bonferroni posttest in comparison with TGF-β-treated cells. SMMC-7721 and MHCC-97H cells were treated with indicated concentrations of sanguinarine. **b and c** Protein expression of N-cadherin and Vimentin was measured by western blotting. Quantification is show on the right. Data were expressed as mean ± SEM (n = 3). **P* < 0.05, ***P* < 0.01, *** *P* <0.001,**** *P* < 0.0001, one-way ANOVA followed by Bonferroni posttest in comparison with control. **d and e** Scratch assay was performed to test the effect of sanguinarine on cell migration. Scale bars, 200 μm. The results shown were representative of 3 independent experiments.


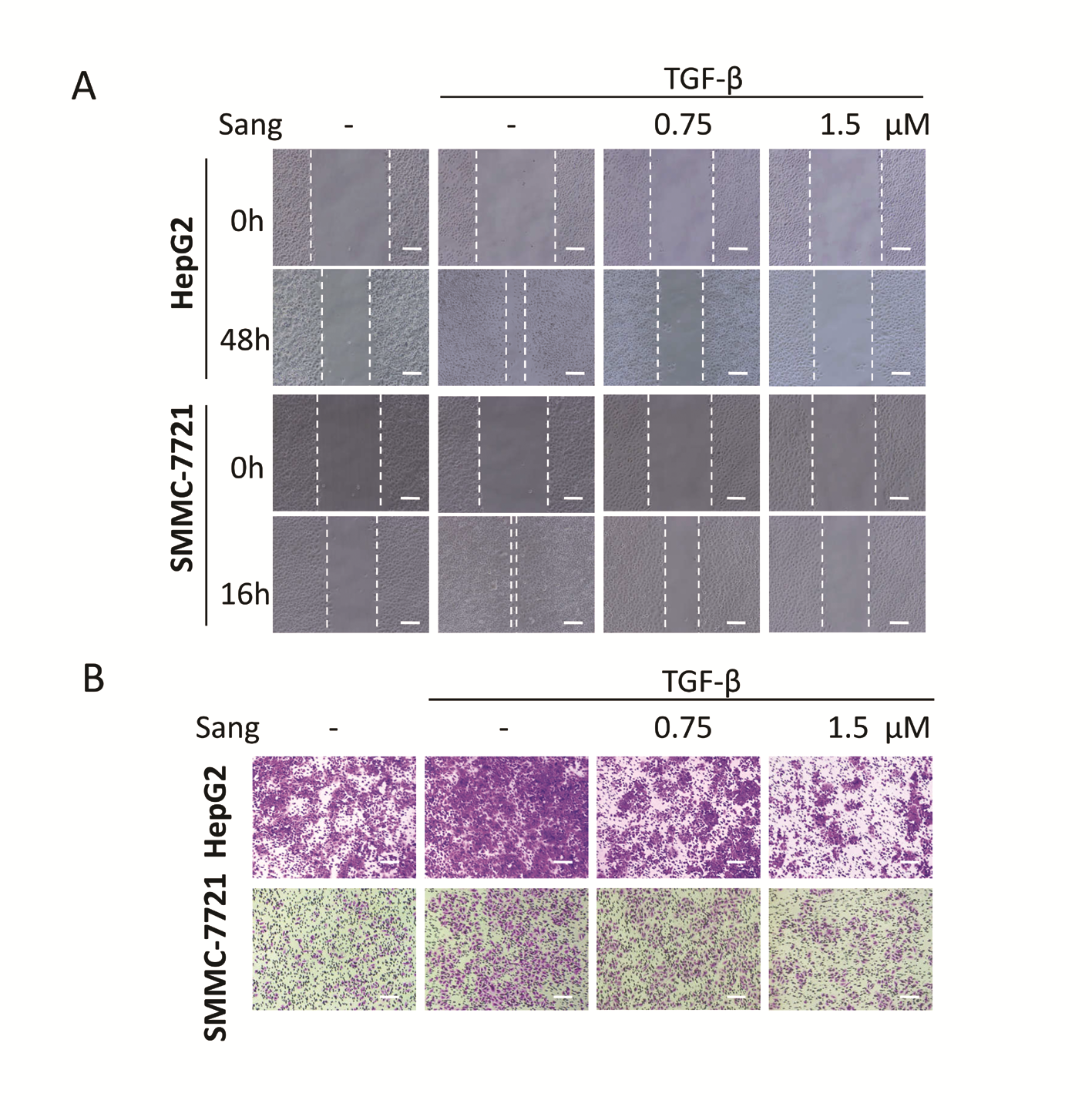


**Supplementary Fig. S5.** Sanguinarine inhibited TGF-β-induced HCC cell migration. **a, b** HepG2 and SMMC-7721 cells were treated with indicated concentrations of sanguinarine in the absence or presence of 10 ng/mL TGF-β. Scratch assay and transwell migration assay were performed to test the effect of sanguinarine on HCC cell migration. Photographs of migrated cells in the scratch assay in the transwell assay were taken at indicated time points. Migrated and 0.2% crystal violet stained cells were photographed after 48 h stimulation and 24 h migration. Scale bars, 200 μm.
